# Novel Mobile and Facility Reproductive Health Kits: enhancing safe abortion and post-abortion care during disasters and humanitarian crises

**DOI:** 10.1186/s12978-026-02419-5

**Published:** 2026-07-30

**Authors:** Nibedita S. Ray-Bennett, Elvina Mustary, Te Klangboonkrong, Shahidul Hoque, Maqbul Bhuiyan, Fariha Haseen, Adewumi Otonne, Lasith Dissanayake

**Affiliations:** 1 School of Business and Institute for Environmental Futures, University of Leicester, Leicester, UK; 2 Reproductive Health Services Training and Education Program (RHSTEP), Dhaka, Bangladesh; 3 Avoidable Deaths Network, Institute for Environmental Futures, University of Leicester, Leicester, UK; 4 School of Global Studies, Thammasat University, Bangkok, Thailand; 5 Data Management Aid, Dhaka, Bangladesh; 6 Avoidable Deaths Network, Bangladesh Chapter, Dhaka, Bangladesh; 7 Department of Public Health & Informatics, Bangladesh Medical University, Dhaka, Bangladesh; 8 Faculty of Life Sciences and Education, University of South Wales, Pontypridd, UK

**Keywords:** Bangladesh, Crises, Disasters, Facility and Mobile Reproductive Health Kits, Health system resilience, Unsafe abortion, Post-abortion care (PAC), Low-and middle-income countries (LMICs)

## Abstract

**Background:**

Maternal deaths from unsafe abortion and post-abortion complications are entirely preventable. Yet, women and adolescent girls in low- and middle-income countries (LMICs) continue to die due to a lack of timely, quality care, especially during disasters. Access to safe abortion and post-abortion care (PAC) is promoted through several strategies, including the Inter-Agency Reproductive Health (IARH) Kit 8. This study introduces the findings of two novel RH Kits prototypes, Facility and Mobile for disasters and crises, exploring: (i) the estimated number of affected persons a combined Kit can serve; (ii) the total and itemised cost of producing these Kits; and (iii) how the prototype Facility Kit aligns with national safe abortion aka menstrual regulation (MR) and PAC guidelines in Bangladesh compared to Kit 8.

**Methods:**

A mixed-methods explanatory sequential design was used to prototype the Kits, combining document reviews, survey findings, key informant consultations, a focus group discussion, and expert input (Jan 2024–July 2025). Data were analysed using descriptive statistics, side-by-side analysis and triangulation.

**Results:**

Findings indicate the Kits can serve 5–30 affected persons, depending on facility capacity. Estimated costs range from USD 535 (small Facility Kit) to USD 1,178 (large Facility Kit) and USD 82 (Mobile Kit). The Facility Kit offers a comprehensive, locally adapted package, including sterilisation equipment and context-specific design.

**Conclusions:**

These Kits have the potential to strengthen health system resilience to disasters. The Mobile Kit has the potential to address last-mile delivery, enabling trained providers to reach women in hard-to-reach areas affected by disasters. This innovation can improve access to safe abortion and PAC during disasters and can offer a scalable model for Bangladesh and other LMICs facing recurrent climate-related hazards.

## Background

Deaths from unsafe abortion and post-abortion complications are avoidable. Yet every day, women and adolescent girls die. In 2020 alone, according to the World Health Organization [1], almost 800 women die every day from preventable causes related to pregnancy and childbirth. In 2024, the global incidence of induced abortion was estimated at approximately 73 million annually, accounting for about 61% of the 121 million unintended pregnancies each year from 2015 [1]. Of these abortions, roughly 45% were considered unsafe, with the vast majority (around 97%) occurring in low- and middle-income countries (LMICs) [1, 2].

Deaths from unsafe abortion and post-abortion complications occur due to multiple factors, including (although not limited to) health system failures, social determinants, harmful gender norms and or inequalities, resulting in a low prioritisation of the rights of women and girls for safe, quality and affordable SRH services. These deaths are also driven by stock-outs of medicines and supplies, as well as external factors that contribute to instability and health system fragility, such as climate change, *extreme weather events* [our emphasis], and humanitarian crises [1, 3]. According to the Global Humanitarian Overview 2024, nearly 300 million people worldwide required humanitarian assistance in 2023 alone, driven by conflict, climate-related disasters, and economic factors, among other causes [4]. Climate-related disasters and conflicts worsen maternal health risks by disrupting access to timely SRH services, including post-abortion care (PAC).

While comprehensive data related to maternal mortality and morbidity during disasters and crises are scarce, the United Nations Population Fund [5], estimated that the number of maternal deaths in the 35 countries that were affected by humanitarian crises or fragile conditions in 2015 was 185,000, representing 61% of the global estimate of maternal deaths (303,000) and 1.9 times higher than the global estimated maternal mortality ratio of 216 per 100,000 live births [5]. Climate-related disasters such as floods, cyclones and storms damage health infrastructure, interrupt supply chains for essential reproductive health commodities, and limit access to family planning and safe abortion services, increasing the likelihood of unintended pregnancies and unsafe abortion practices. In Nepal, the 2015 Gorkha earthquake affected over 1.4 million women of reproductive age and severely damaged health facilities, resulting in reduced access to antenatal, delivery, and emergency obstetric care in the most affected districts [6]. Similarly, in Nigeria, large-scale flooding led to population displacement and damage to health infrastructure, disrupting access to antenatal care, skilled birth attendance, and essential maternal health services in affected areas [7]. In low-resource and disaster-affected settings, these disruptions translate into reduced use of antenatal and postnatal care and increased vulnerability to pregnancy complications, ultimately contributing to higher maternal morbidity and mortality [8, 9].

### Intervention: RH kits and RHCC

Reducing maternal mortality and morbidity is currently part of the UN’s Sustainable Development Goal (SDG) 3 (Good Health and Well-Being) and Target 3.1 (reduce global mortality ratio to less than 70 per 100,000 live births) by 2030. Reducing maternal mortality and morbidity associated with unsafe abortion and PAC requires a range of interventions. These include training community health workers, pharmacy staff, and pharmacists to provide medical abortion management up to 12 weeks of gestation [10]; developing quality care guidelines to support the self-management of medical abortion; promoting awareness about contraception through sexuality education; ensuring access to safe and legal induced abortion; providing timely treatment for complications [1], and offering abortion counselling via telephone [11]. Beyond these measures, this study highlights the use of Reproductive Health Kits (RH kits) as an additional intervention strategy to strengthen responses to unsafe abortion, safe abortion (*aka* menstrual regulation (MR) in Bangladesh), and PAC, specifically during disasters and humanitarian crises.

Notably, a global systematic review conducted by the lead author covering the period 2010–2022 identified no localised RH kits for PAC, MR, safe abortion, apart from the Inter-Agency Reproductive Health (IARH) Kit 8 [3].

The IARH Kits are standardised, pre-packaged sets of life-saving medicines, equipment, and supplies designed to support the delivery of the Minimum Initial Service Package (MISP) during humanitarian emergencies [12]. There are 13 IARH Kits, designed to provide all services included in the MISP for a 3-month period and maintained by UNFPA globally.

Given this study’s focus on the prototype development of a facility-level RH kit and a complementary portable kit, IARH Kits are key to the design as they are the global standard. Specific to MR and post-abortion complications, IARH Kits 6 (Clinical delivery assistance) and 8 (Management of complications of miscarriage or abortion) are of particular interest to this study as they provide a benchmark for potential localisation and adaptation. Both Kits are designed for primary health-care facility level and for use by “trained health-care providers with additional midwifery and selected obstetric and neonatal skills at the health centre or hospital level” ( [12], p. 23). Other IARH Kits may be used by trained service providers at the community health-care level, such as Kit 2 (Clean delivery) or administration of SRH treatment (Kits 1 A, 3, 4, 5). IARH Kits designed for referral hospital use in surgical obstetrics are of less relevance to the scope of this study. At present, no equivalent emergency-oriented kits are readily available on the global market [3].

RH kits are often positioned and administered during natural hazard disasters and humanitarian crises to reduce maternal mortality and morbidity [12, 13]. In the absence of or limited access to safe, effective, affordable, and acceptable methods of reproductive health services, RH kits play a vital role in service continuity, especially for the most marginalised and vulnerable segments of the population in LMICs. They ensure the dignity of vulnerable women and adolescent girls of reproductive age caught up in refugee camps or cyclone-cum-flood shelters. RH kits are available in single- or multi-provider packages that include essential medicines and supplies to speed the provision of appropriate RH services [12–15].

In 2021, Ray-Bennett et al. [16], identified 36 distinct RH-related kits worldwide, including the IARH kits. Of which, only two kits are designed for the treatment of safe abortion and PAC-related complications, viz. IARH Kit 8 and the Sterimed Group’s Manual Vacuum Aspiration (MVA) Kit [15, 16]. The IARH Kit 8 is specifically designed to “treat the complications arising from miscarriage (spontaneous abortion) and unsafe induced abortion, including sepsis, incomplete evacuation and bleeding” during disasters and emergencies for three months ( [14], p.27). The Sterimed Group’s Kit is designed to assist with an incomplete miscarriage or the medical termination of a pregnancy. The IARH Kit 8 is not available nationally, whereas the MVA Kits are, and they can be procured from Sterimed Group or Eurosurgical. Although MVA Kits are available, they are not explicitly designed for disasters and humanitarian crises [16].

To address this critical gap, the lead author at the University of Leicester, in collaboration with Bangabandhu Sheikh Mujib Medical University (currently known as Bangladesh Medical University), Data Management Aid, and the Government of Bangladesh’s Directorate General of Family Planning (DGFP), and with funding from the International Planned Parenthood Federation – South Asia Region Office (IPPF-SARO) and the Economic and Social Research Council (ESRC), developed a protocol for two novel localised RH Kits: the Facility RH Kit and the Crisis RH Kit (renamed as Mobile Kit) [15]. The Kits protocol included a comprehensive list of i) essential and emergency drugs, ii) equipment, iii) consumable supplies, and iv) information and education materials. This protocol was developed in consultation with the DGFP and the Obstetric and Gynaecological Society of Bangladesh, and involved systematic reviews, stakeholder workshops, and technical consultations between 2020 and 2021 [15]. Tables 1 and 2 provide an overview of the Facility RH Kit and the Mobile RH Kit components.

**Table 1:** Summary of contents of the Facility Kit – PAC, PAC & MR/Safe Abortion

Components	Content - PAC	Content - PAC & MR
Medicines	• PAC management • Post-MR and PAC contraceptives • Antibiotics and pain management (according to the national guidelines) • Emergency drugs • Disinfectant • Topical use medicines • Supplements	• MRM Kit (Menstrual Regulation Drugs – Combination of Mifepristone and Misoprostol according to the national guidelines) • Post-MR and PAC management • PAC contraceptives • Antibiotics and pain management (according to the national guidelines) • Emergency drugs • Disinfectant • Topical use medicines • Supplements
Equipment	• MVA set • Sterilisation items • Instruments • Personal protective equipment (PPE) • Other equipment	• MVA set • Sterilisation items • Instruments • Personal protective equipment (PPE) • Other equipment
Consumable supplies	• Other consumable supplies	• Other consumable supplies
Information and educational materials	• Material for post-procedure patient leaflet on MR, MR and PAC • Instruction guidelines • Record-keeping document and complaint form	• Material for post-procedure patient leaflet on MR, MR and PAC • Instruction guidelines • Record-keeping document and complaint form

Source: Ray-Bennett et al. [16] and prototype engagement data

**Table 2:** Summary of contents of the Mobile Kit

Components	Content
Medicines	• MRM Kit (Menstrual Regulation Drugs – Combination of Mifepristone and Misoprostol according to the national guidelines) • PAC management • Post MR and PAC contraceptives • Pain management (according to the national guidelines) • Topical use medicines • Supplements
Equipment	• Blood pressure monitor with stethoscope
Consumable supplies	• Other consumable supplies • Personal Protective Equipment (PPE) • Other equipment (torch light)
Information and educational materials	• Material for post-procedure patient leaflet on MR, MR and PAC • Instruction guidelines • Record-keeping document

Source: Ray-Bennett et al. [16] and prototype engagement data

The Facility and the Mobile RH Kits are designed to treat incomplete abortion, missed abortion, and PAC management (before and after 12 weeks of missed period of menstruation) during disasters and humanitarian crises in Bangladesh. The novelty of these Kits is that they are localised to align with Bangladesh’s Comprehensive MR and PAC Service Guidelines and the broader SRH needs of women and adolescent girls in disaster-prone rural areas and hard-to-reach health facilities in Bangladesh. These Kits were prototyped between August 2023 and March 2025 by the lead author at the University of Leicester, in collaboration with Data Management Aid, the DGFP, and a new partner, the Reproductive Health Services Training and Education Program (RHSTEP), with funding from ESRC-IAA and ESRC-Commercialisation funds. This article presents the main findings from the prototype phase and the user-engagement activities undertaken in July 2025, funded by the UKRI–ESRC SHAPE Catalyst Fund. The research questions (RQs) that guided the prototype phase were:

1. What is the estimated number of affected persons a single Facility and Mobile Kit prototype combination can effectively serve during a disaster scenario in Bangladesh in general and in the pilot site in particular?
2. What is the estimated total and itemised unit cost required to produce one complete Facility and Mobile Kit prototype, based on current market rates for its components in Bangladesh?
3. To what extent does the localised item composition of the prototype Facility Kit better align with the national safe abortion or MR and PAC guidelines and specific primary healthcare needs in hard-to-reach, disaster-prone areas in Bangladesh compared to the standard IARH Kit 8?

The research questions were addressed using a combination of primary and secondary research methods, complemented by stakeholder engagement activities, as detailed in the Methodology section. The following section provides the Bangladesh context and study approach that underpinned the development of the Facility and Mobile Kits for disasters and crises. Subsequently, the research methods, results, and discussion are presented along with a few key recommendations in the concluding section.

## Bangladesh: disasters, hard-to-reach areas, health systems

Bangladesh is highly exposed to natural hazards and climate risks due to its geographic location and the effects of global warming. The majority of its land area comprises a low-lying river delta formed by the three major river basins, with more than 230 rivers, thousands of tributaries, and numerous canals [17]. Its coastal position along the Bay of Bengal further heightens vulnerability to tropical cyclones. The 5th and 6th IPCC Reports highlight that climate change impacts are already severe, manifesting in the loss of agricultural land, rising homelessness and displacement, and mounting pressure on health systems and infrastructure [18–20]. These challenges are particularly acute in rural, remote, and hard-to-reach areas of Bangladesh [21, 22]. Hard-to-reach areas are defined “in terms of both their remote geographical location, as well as the population residing out of the range of development activities”, and the people residing in these areas are called *hard-to-reach people* due to their social exclusion from adequate health and other necessities ( [23], p.1). Bangladesh has several hard-to-reach areas, including *char* (or newly emerged land strips along riverbanks), haor (or backswamp), coastal areas, hill tracts, and tea gardens. These challenging geological conditions expose them to frequent natural hazard events [23].

In 2015, the Sendai Framework for Disaster Risk Reduction (hereafter, Sendai Framework) urged all UN Member States to ‘build the resilience of health systems’ as part of its Global Target D by 2030 [24]. Although Target D is not prescriptive about which tier of health care should be the focus of disaster resilience, this study emphasises supporting resilience at the PHC level. In Bangladesh, the first point of contact for the rural population in the health system is the PHC facilities, which include Community Clinics at the village level; Health and Family Welfare Centres and Mother and Child Welfare Centres at the Union level; and Upazila Health Complexes at the sub-district level [24, 25]. The Union Health and Family Welfare Centres, Mother and Child Welfare Centres, and Upazila Health Complex facilities fulfil roles similar to those of District Hospitals for SRH [26], including PAC and MR [27].

Studies in Bangladesh and elsewhere have also confirmed the vital role that PHCs play during floods [26], as well as in reducing mortality and morbidity through the provisions of family planning services, contraception and basic essential health care services [24, 28, 29].

Despite these achievements, concerns remain regarding the outreach of PHC services in rural Bangladesh [30], particularly in hard-to-reach areas [27]. PHCs are chronically underfunded, with facilities inadequately stocked with medical instruments and supplies [24], and understaffed with skilled personnel [27, 31]. A resilient PHC system should sustain essential services, including reproductive health, during disasters with minimal disruption. Yet, building resilience is challenging in contexts of overstretched staff and weak governance. As Koblinsky (2016) ( [32], p.23) argue:

“Resilience demands mechanisms to ensure essential health services are delivered, regardless of the stress on the system; and must include the capacity to address the special needs of women, adolescents […] by increasing and optimising the health workforce and improving facility capability.”

While strengthening the health workforce and upgrading facilities requires multi-actor interventions and sustained financial commitment from the Government of Bangladesh and international donors, in the context of this study, it is argued that the Facility and Mobile Kits offer a pragmatic means to enhance PHC capacity for disaster readiness and preparedness for safe abortion/MR and PAC.

Health systems are understood as all individuals, institutions, and activities primarily aimed at improving, preserving or restoring health [33]. In Bangladesh, the health system is pluralistic because MR and PAC services, along with other services, are delivered by many different providers, including PHCs, private providers, public providers, and informal providers [34, 35]. Private clinics in rural areas are few and far between for SRH services, including MR and PAC. Hence, rural women and girls of reproductive age rely heavily on pharmacies and informal providers such as drug sellers and traditional birth attendants, who are often untrained [35, 36].

Also, frequent flooding disrupts access to healthcare. For example, in one flood-prone riverine island, 79.4% of women gave birth at home, and 6% of those home deliveries resulted in adverse outcomes, including infant death, when health facilities were unreachable [37]. An assessment in climate-vulnerable northeast Bangladesh found that 52% of surveyed health facilities had to close during floods [38]. Likewise, on average, Community Clinics were closed for eight days, with closures ranging from as short as one day to as long as 60 days [38]. Such disruption leaves women with no choice but to rely on unsafe deliveries and choices.

As part of this pluralistic model, Bangladesh's health financing is characterised by heavy reliance on out-of-pocket payments and high out-of-pocket expenditure, with no universal government health insurance scheme [35, 39]. Although MR and PAC services at PHCs are designated as free under Bangladesh's national family planning program, households bear approximately 68.5% of total health expenditure while the government covers only about 26% (these figures reflect general health spending rather than MR/PAC services specifically).

Health insurance remains largely absent in rural areas [40], and the inequitable distribution of government health financing places a disproportionate financial burden on low-income households seeking care [41]. Consequently, many patients, including wealthier households, turn to private providers with higher costs or incur high out-of-pocket costs even in public facilities [41, 42]. The absence of comprehensive insurance compels rural women, particularly in hard-to-reach and climate-vulnerable regions, to pay most SRH expenses out-of-pocket, pushing them towards informal providers who are often cheaper than public facilities once transport and informal fees are considered, thereby deepening inequalities in access to MR and PAC services [41, 42].

Our localised prototype Facility and Mobile RH Kits are designed to reduce inequalities in access to essential services. These Kits aim to strengthen PHCs in hard-to-reach and climate-prone areas by directly addressing service delivery gaps and reducing reliance on informal providers. They aim to do so by: i) bringing services closer to communities; ii) empowering frontline providers; iii) ensuring service availability during disasters; iv) supplying essential and emergency drugs, equipment, and consumables; and v) preventing maternal deaths and morbidity. By equipping service providers with the tools and supplies needed for MR and PAC, the Kits and associated training and capacity-building under the RHCC package have the potential to ensure that more women receive appropriate care, ultimately saving lives.

**Fig. 1:**
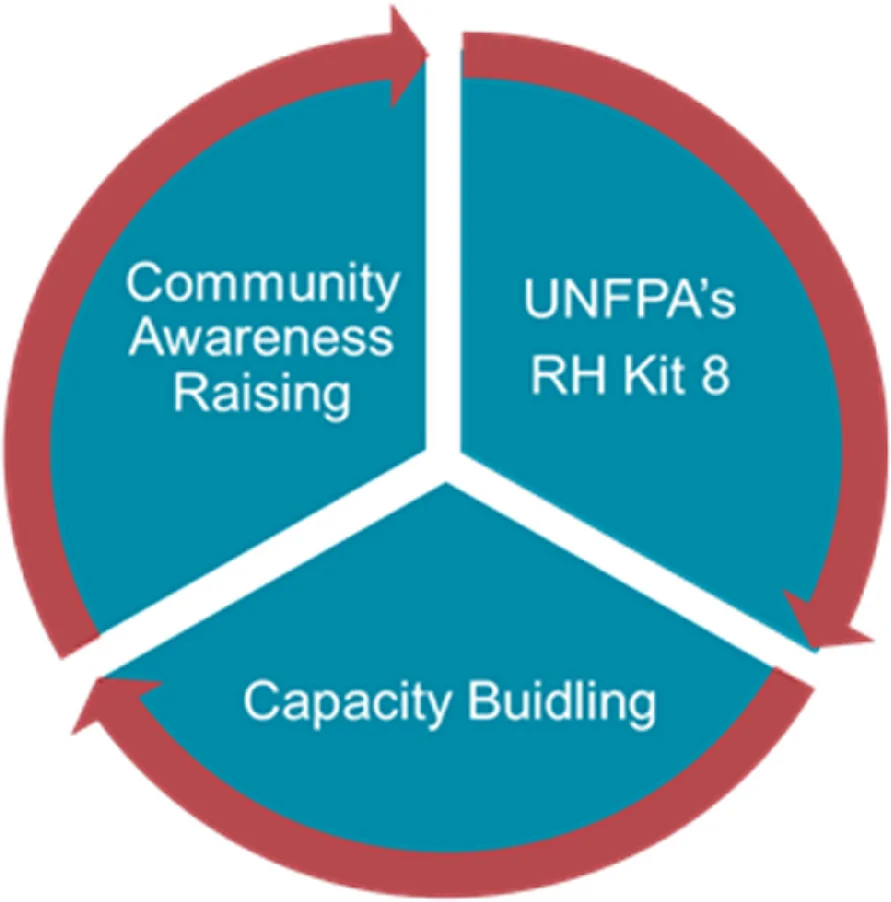
The RHCC Intervention. Source: Ray-Bennett et al. [13]

The Facility and Mobile Kits were developed as part of an evidence-based implementation research intervention known as the RHCC package in flood-prone Belkuchi Upazila of Sirajganj District, Bangladesh. This package consisted of three components: pre-positioning IARH Kit 8, Capacity building of health professionals, and Community awareness-raising. The RHCC intervention was piloted from 2015 to 2018 across five flood-prone primary health care (PHC) facilities in Belkuchi Upazila (sub-district) [13] (see Fig. 1).

As part of this intervention, 26% of service providers who needed MR and PAC training (one medical officer, three nurses, and six family welfare visitors) in the selected PHCs received training to administer the IARH Kit 8. The IARH Kits 8 were procured from Copenhagen, Denmark, in 2016, and complemented by community-level awareness activities. During the 2017 floods, 48 women directly benefited from this demand-led intervention, demonstrating its potential to strengthen RH service delivery in disaster contexts.

The trial of the IARH Kit 8, together with other components of the RHCC intervention, was found to have directly enhanced service providers’ skills and knowledge in safe abortion or MR and PAC, family planning counselling, record keeping, quality of care delivered to women clients/beneficiaries, and importantly, the availability of PAC for the first time in the selected PHCs during the 2017 floods in Belkuchi Upazila [13, 22]. Despite these evident benefits, several challenges emerged regarding the feasibility of the IARH Kit 8 in disaster-prone LMICs such as Bangladesh. Procurement from UNFPA in Copenhagen, Denmark, was costly and had long lead times. In addition, some medicines and equipment included in the Kit did not comply with the Government of Bangladesh's national guidelines. For example, the dilatation and curettage set and misoprostol tablets were incompatible with national guidelines on MR and PAC. A key recommendation from the RHCC project was to use the IARH Kit template and to develop a nationally relevant MR and PAC RH Kit tailored to the specific local needs of women and adolescents, the health system and the contexts of local disasters and crises.

In response, new protocols for two complementary disaster kits, the Facility RH Kit and the Mobile RH Kit, were developed between 2020 and 2021 [15], and were subsequently prototyped in Bangladesh between 2023 and 2025 by the lead author and in-country collaborators, as mentioned in Sect. "Background". The next section outlines the research methodology and data-collection procedures used to address the three research questions for the prototype phase.

## Methodology

### Study design and sources of data

To prototype the Facility and Mobile RH Kits, a mixed-methods explanatory sequential design was employed [43, 44]. In the first phase, quantitative results informed the design of the second and third phases, which involved further quantitative data collection followed by qualitative data collection, respectively. Data collection methods included document review (e.g., national statistics, web reports, and MR and PAC services guidelines), statistical analysis, a survey, key informant consultations, a focus group discussion (FGD), and expert consultations from January 2024 to July 2025. The details of these data collection methods are as follows.

### Phase 1: document review, study site, statistical analysis and survey

Based on the review of the *Country Climate and Development Report* [45], *the Climate Risk Country File Projections* [46]*, and the Multi-Hazard Risk Analysis: Climate-Related Disasters* [47], the study site was selected. According to the World Bank Group report, Bangladesh ranked seventh globally in the ‘2021 World Climate Risk Index’ [48]. This Index also ranked 64 districts from highest to lowest in terms of vulnerability to natural hazard disasters across Bangladesh's eight divisions. Four districts are marked ‘very high’, and another 27 districts are marked as ‘high’ for climate-related multi-hazard risk [49] (see Table 3). To estimate the need for the Facility Kit, a subset of 21 out of four ‘very high’ and 27 ‘high-risk’ Districts (61%) were chosen for this study. Of the 27 high-risk Districts, Cox’s Bazar District in the Chittagong Division was selected as the study site for data collection.^1^

**Table 3:** Number of districts in climate-related multi-hazard risk

Risk level	Number of Districts	District %
Very high	4	6.25
High	27	42.19
Medium	21	32.81
Low	12	18.75
Total	64	100

Source: Mita et. al. ( [49], p. 26)

The Districts of Bangladesh vary in topography, the types of hazards residents face, and the capacity of PHC facilities. To capture this diversity, Cox’s Bazar district was selected as the study site because it encompasses all three topographies: plain, coastal, and hill tract. Moreover, the district experienced four major disasters in recent years: Cyclone Mocha (2023), Cyclone Sitrang (2022), Cyclone Hamoon (2023), and floods (2023). This recent exposure to disasters made Cox’s Bazar an appropriate site for data collection for the survey, key informant consultants and FGDs, as it helped minimise recall bias. Therefore, Cox’s Bazar was an ideal case study site due to its high multi-hazard climate risk, diverse topography, and experience of four major disasters between 2022 and 2024, when the study began, enabling prototype testing and evidence generation for RQ1.

#### Survey

After selecting the study site based on the reasons mentioned above, through word of mouth and field visits by Data Management Aid, 458 cyclone/flood emergency shelters were identified in Cox’s Bazar across hilly, coastal, and plain areas. Using WHO’s cluster sampling design [50, 51], a representative sample of 30 emergency shelters was selected across three topographies. The survey questions focused on four topics with 29 closed-ended questions: i) details of the cyclone/flood shelter; ii) the capacity and utilisation of the shelter, ii) the provision of RH care during disasters, and iii) assessing the feasibility of administering the Mobile RH Kit by the Union Health and Family Welfare Centres during or after disasters. The questionnaire was translated into Bangla by Data Management Aid.

The surveys were conducted between March and April 2024 by four experienced local data collectors from a Bangladesh-based research partner, Data Management Aid. All the data collectors attended a day-long training on the survey procedure and ethics protocol. Surveys were conducted face-to-face with representatives of 30 shelters under the guidance of a senior supervisor based in Cox’s Bazar. Data were collected using tablets. The Open Data Kit-based tools incorporated robust validation and logical checks. Data was stored in real time on a cloud-based, secure server. The senior supervisor ensured the quality of the collected data by cross-checking a few samples. These survey data were analysed using descriptive analysis (historical data, frequencies, mean, and percentages) conducted in Statistical Package for the Social Sciences (SPSS) software [52, 53], to determine i) the estimated number of affected persons served by each Mobile Kit (RQ1).

### Phase 2: document review for projections and cost estimates

The study also drew on multiple secondary data sources to assess PAC service needs across 21 ‘very high’ and ‘high’ climate-related multi-hazard risk selected districts (Phase 1 – see above) to estimate the number of affected persons a single Facility and Mobile Kit prototype combination can effectively serve (RQ1).

Data were obtained from national population censuses, population projections produced by the Bangladesh Bureau of Statistics, and government-endorsed reproductive health and MR and PAC services guidelines (see Table 4). Additional sources included climate risk assessments and vulnerability indices developed by national and international organisations to capture patterns of exposure to climate-related hazards across districts, as mentioned in Phase 1.

**Table 4:** Documents Reviewed

National census statistics	*Population and Housing Census* (2015)
Population projections	*Population Projection* (2022)
Policy guideline	*Comprehensive MR and PAC Services Guidelines* (2021)

A structured, stepwise estimation framework was applied to secondary demographic and health data to characterise population dynamics relevant to reproductive health planning. This included analysis of population size, age and sex distribution, and reproductive age cohorts, followed by the application of nationally accepted reproductive health indicators drawn from the Comprehensive MR and PAC Services Guideline documents. Secondary data were systematically synthesised to inform assumptions regarding potential service needs during disaster periods and to support the design parameters of the Facility and Mobile RH Kits.

All analyses were based on publicly available or government-authorised data sources to ensure consistency with the MR and PAC Guidelines. Based on the Population Projection data available in 2024, the projection increased year on year with the birthrate of Bangladesh around 1.25% and used a top-down approach to get the proportion of reproductive age women population in Bangladesh and then multiplied the number by the current maternal mortality rate to arrive at the expected post-abortion complication cases.

#### Cost estimates

To address RQ 2, the estimated price for the Facility and Mobile Kits was determined from two sources. One source was estimated by the health economist and financial analyst consultant of Data Management Aid based on unit prices from the open market, while another price source was included from our supplier, RHSTEP, who prototyped one large-sized box of the Facility Kit (MR and PAC), one Mobile Kit and one portable backpack.

The content price sourced from the open market was determined based on the current market prices, including any value-added taxes required for procurement under the government or institutional framework. The source of the price points included physical visits to Model Pharmacies in Dhaka and BMA (Bangladesh Medical Association) Bhaban in Paltan, Dhaka, from March to May 2024. The prices of medicines and consumable supplies were collected from pharmacies at the Malibagh Medicine Wholesale Market in Dhaka and from e-pharmacies. The prices of consumable supplies were also obtained from the online portals of departmental stores such as Shwapno^2^ and Chaldal^3^ in 2024.

### Phase 3: key informant consultations and FGD

In addition to the statistical analysis for RQ 1 (mentioned above), the estimates were also iteratively refined through key informant consultations, FGD and ongoing consultation with the Technical Advisory Group (TAG) to reflect facility-level capacity, service delivery constraints during disasters, and patterns of population displacement in the study site. They are discussed here and in the next section.

In April 2024, the consultant economist and financial analysts from the Data Management Aid conducted five key informant consultations and one FGD in Cox’s Bazar. The consultations were conducted with executive members of two cyclone shelters and one each with the Deputy Civil Surgeon and the Deputy Director of Family Planning. These purposive consultations were conducted to gather opinions on the volume of medicines required for the Mobile Kit (RQ1) and to triangulate qualitative insights with survey data and document reviews. Each consultation lasted approximately 20–30 min and was conducted in Bangla. The notes of the consultations were retained by the financial analyst. These qualitative insights complemented responses to two questions included in the survey questionnaire:

- 209: How long did the evacuees stay at the shelter during the most recent disaster?
- 405: Are any FWV/FWA/Midwives deployed in the shelter during disasters?

The FGD consisted of 12 participants at Cox’s Bazar Municipality. Of these, seven were health personnel, and five were health administrators. This purposive FGD sought to capture users’ and health managers’ perspectives on the preconditions for positioning the Mobile Kit and the required volume of medicines (RQ1). The FGD was conducted in Bangla and lasted approximately 50–60 min.

Qualitative inputs from health personnel and cyclone shelter staff were used to inform key assumptions regarding service demand and operational feasibility. These inputs were particularly important given that flood-cum-cyclone and emergency shelter managers do not routinely maintain records on evacuee numbers or gender composition. In the absence of reliable administrative data, experiential insights from frontline personnel were therefore treated as a critical source of contextual evidence. These qualitative inputs were systematically incorporated into the design process of the Facility and Mobile RH Kits, informing the development of modular units with multiple size options for the Facility Kit to support flexible deployment across varying service contexts, facility capacities, and disaster scenarios.

#### Facility Kit and IARH Kit 8 comparison

To address RQ 3, a comparative analysis was conducted between the Facility Kit and the IARH Kit 8. The comparison was structured around six dimensions: use cases, procurement and logistics, flexibility, regulatory compliance, adaptability and value for money. Data for the comparison were primarily drawn from the *Inter-Agency Reproductive Health Kits for Crisis Situations Manuals* (Sixth Edition, 2019—the most recent edition available during the study period—and the Fifth Edition, 2011) [12, 14], with particular attention to the specifications and contents of IARH Kit 8. As pricing information for the IARH Kit 8 was not publicly available, the first author contacted the UNFPA Procurement Services Branch and Humanitarian-SRH Supplies by email in May 2025. The confirmed unit price at that time was USD 1,139.41. Additional pricing data were drawn from the lead author’s prior research conducted in 2017, during which four IARH Kits 8 were procured from Copenhagen and transported to Dhaka for implementation in Sirajganj District, Bangladesh.

At that time, the cost per IARH Kit 8 was approximately USD 1,344 (BDT 159,557), with each Kit designed to serve 60 affected individuals. Storage in a rented warehouse was required to maintain cold-chain conditions for oxytocin. The Kits were subsequently transported to Belkuchi Upazila and pre-positioned in four health facilities ahead of the monsoon season. Additional implementation costs, including training for 10 healthcare professionals and 3 community awareness events, amounted to USD 90,745 [27].

These cost and specification data, together with information extracted from the UNFPA Manuals, were systematically analysed and compared with the Facility Kit. The findings of this comparative analysis are presented in tabular form (see Table 12) in the Results section.

#### Expert consultations

Technical and policy experts with established track records in SRH in Bangladesh and/or internationally were regularly engaged throughout the project’s duration, starting from Phase 1. They scrutinised the research's assumptions, provided expert opinions on the prototype’s feasibility in Bangladesh, and evaluated the merits of the Facility and Mobile Kits against comparable solutions. The engagement took place in two formats: the Technical Advisory Group (TAG) and the Dissemination and Approval Panels.

TAG consisted of 13 experts from various types of organisations, including the DGFP, humanitarian and UN organisations (e.g. UNFPA-Country Office, IPPF-Humanitarian Office), NGOs (Ipas-Bangladesh), universities (Dhaka and Bangladesh Medical Universities), pharmaceutical companies, and a social enterprise specialising in SRH. Four virtual 1–1.5-h meetings were held quarterly from November 2023 to July 2024. Although not all TAG members were available for the entire duration of all meetings, they were also engaged individually to ensure members were kept informed and had opportunities to provide feedback or suggestions outside formally scheduled meetings.

Members of the TAG played a vital role in shaping the development of the Kits, including recommending renaming the Crisis Kit to the Mobile Kit, benchmarking the Facility Kit against the IARH Kit 8, and introducing modular flexibility to the Facility Kit, among other contributions. The Kit protocol [15], was also updated to include a fourth component, “Educational Materials,” comprising instructional guidelines and IEC materials. In addition, two manuals were developed for each Kit, alongside a research brief. These documents were reviewed by four of the 13 experts and, following peer review, were presented to the Approval Panels in Phase 4.

### Phase 4: dissemination and approval panels and user engagement

Upon completion of data collection and peer review, the findings were presented to a panel of experts at the DGFP Office in Dhaka for review and feedback. The standard protocol of the DGFP Office was followed to request a panel meeting (e.g., submitting a formal letter to the DGFP Office, awaiting approval, and confirmation of the meeting date, among other steps). The findings were presented to the Panel in the form of a research brief, manuals, instruction guidelines, and PowerPoint slides detailing the content of the Kits. The panel was convened by the DGFP Office in Dhaka, in coordination with the project partner lead in Bangladesh (RHSTEP). Fourteen members attended the Panel: ten officials from the DGFP, one from the Directorate General of Health Services, and one each from CARE-Bangladesh, Ipas, and Bangladesh Medical University. All the project investigators attended this Panel meeting. A DGFP representative moderated the workshop under the chairmanship of the Director of Mother and Child Health Services on 19 November 2024. The Expert Panel recommended five revisions to the content list, and the project team accepted these revisions:

- Minor changes in wording for improved clarity;
- Removal of oxytocin, sterilising drum, and autoclave tape from the equipment list;
- Definition of ‘emergency drug’ in the content list;
- Removal of Bangla versions of record-keeping documents and the complaint form from the IEC list; and
- Addition of a client information sheet to the IEC list.

On 28 June 2025, another consultation was arranged with three users or health personnel at the Uttarkhan Union Health & Family Welfare Centre in Dhaka District to obtain feedback on the Mobile Kit packaging. During this consultation, the inclusion of an additional drug for cramp management in the Kits was suggested. On 29 June 2025, this recommendation was reviewed and approved at a subsequent Expert Panel meeting attended by six DGFP personnel, moderated by the same DGFP representative under the chairmanship of the Director of the Mother and Child Health Services. All the project investigators attended this Expert Panel meeting. Between June and July, the first author also consulted with Dycine Pharmaceutical Company Ltd. to optimise per-package quantities of the Kit as part of market-readiness planning.

### Data analysis strategy

In this mixed-methods explanatory sequential design, quantitative and qualitative data were analysed separately [43], because data collection occurred across three interconnected phases. Nevertheless, the overall analysis incorporated ‘integration’ strategies of *connecting* and *building* [54]. *Connecting* involved using findings from the initial quantitative phases (document review, study site) to inform purposive sampling for the qualitative phase, whereby a subset of emergency shelter managers from the survey was selected for consultations. *Building* entailed using quantitative findings to shape qualitative data collection tools, including key informant interview guides for consultations with health personnel and engagements with key stakeholders at the DGFP to seek approval of the Kit content, manuals and instructional guidelines. Further consultations with selected service providers in Uttarkhan Union Health and Family Welfare Centre were conducted to refine the Kits’ content and packaging.

An additional integration strategy, triangulation and complementarity of quantitative and qualitative data [55], was employed to address the first two research questions. This approach mitigated methodological limitations, such as the absence of shelter-level records on displaced populations and gender-disaggregated data, one-time cross-sectional survey, and the population-level nature of estimates derived from document reviews. Qualitative insights from study sites, therefore, provided essential contextual detail. Moreover, as the primary users of the Kits are health service providers and potential institutional adopters include the DGFP and the Directorate General of Health Services (DGHS), stakeholder engagement was integral to ensuring localisation, acceptability, and feasibility through triangulation.

Finally, side-by-side displays of quantitative and qualitative findings are a commonly used analytical strategy in explanatory sequential mixed-methods designs [54]. Where appropriate, this study employed two such displays in the Results section (consultation and survey data (see Table 7) and IARH Kit 8 and Facility Kit comparison (see Table 12)) to facilitate integrated interpretation of findings.

### Ethical approval

Ethical approval for this study was obtained in 2024 from the University of Leicester’s Research Ethics Committee (reference: 43,496-nsrb1-ss/bu:m'ing,innov',str'gy&ops,dept) and the University of Dhaka’s Institute for Health Economics Institutional Review Board (reference: HE/IRB/DU/70/2023/Final).

## Results

### Estimated number of affected persons a single Facility and Mobile Kit can serve (RQ1)

To estimate the number of affected persons that a single Facility and Mobile Kit can serve, population estimates for the highly vulnerable districts were analysed and forecast over 10 years, followed by gender distribution and the probable number of menstrual regulation or abortion cases in the population. The population statistics of the selected 21 districts are provided in Table 5.

**Table 5:** District-wise population estimate

District	Population/Year			
	**2022**^a^	2023*	2024*	2032*
Barguna	1,010,530	1,020,743	1,031,060	1,117,434
Bhola	1,932,518	1,952,049	1,971,778	2,136,959
Patuakhali	1,727,254	1,744,711	1,762,344	1,909,980
Bandarban	481,109	485,971	490,883	532,006
Chattogram	9,169,464	9,262,137	9,355,748	10,139,503
Cox's Bazar	2,823,265	2,851,799	2,880,621	3,121,939
Khagrachhari	714,119	721,336	728,627	789,666
Lakshmipur	1,938,111	1,957,699	1,977,485	2,143,144
Noakhali	3,625,252	3,661,892	3,698,901	4,008,768
Rangamati	647,587	654,132	660,743	716,095
Kishoreganj	3,267,630	3,300,655	3,334,014	3,613,313
Shariatpur	1,294,561	1,307,645	1,320,861	1,431,513
Khulna	2,613,385	2,639,798	2,666,478	2,889,855
Satkhira	2,196,581	2,218,781	2,241,206	2,428,958
Jamalpur	2,499,737	2,525,001	2,550,521	2,764,185
Netrokona	2,324,856	2,348,353	2,372,087	2,570,803
Naogaon	2,784,598	2,812,741	2,841,169	3,079,181
Serajganj	3,357,708	3,391,644	3,425,922	3,712,920
Gaibandha	2,562,232	2,588,128	2,614,285	2,833,291
Kurigram	2,329,161	2,352,701	2,376,479	2,575,563
Sunamganj	2,695,495	2,722,738	2,750,256	2,980,652
Total	51,995,153	52,520,655	53,051,468	57,495,728

^a^Bangladesh Bureau of Statistics [56]

^*^Population estimates from 2023 onwards were forecast using the 2022 *Population and Housing Census* data [56], applying population growth ratesreported in *Population Projection of Bangladesh: Dynamics and Trends* [57]

According to census data, 53.94% of the total population fell within the reproductive age group of 15–49 years, with a gender distribution of 47.38% male and 52.62% female; 0.01% identified as belonging to another gender. Based on these statistics, the reproductive-age population across the 21 districts was estimated at 28,329,641 in 2023, of whom 14,907,057 were female. The projected female population of reproductive age was estimated to increase to 15,057,719 in 2024 and 16,319,144 by 2032 across the targeted districts.

The prevailing crude birth rate in Bangladesh was estimated at 17.5 births per 1,000 population, while the proportion of unintended pregnancies was reported at 28.4% [44, 58–60]. According to the *Bangladesh National Comprehensive MR and PAC Services Guidelines* [61], abortions accounted for 29% of all pregnancies, and 61% of unintended pregnancies resulted in abortion. The maternal mortality ratio was estimated at 156 per 100,000 live births, with national targets set to reduce this to 100 per 100,000 by 2025 and 70 per 100,000 by 2032, in line with SDG 3 targets 3.1 and 3.C. Based on these assumptions, the estimated number of women at risk of post-abortion complications was calculated for 2023 and projected for subsequent years across the 21 targeted districts was 193 cases in 2023, 195 cases in 2024, and 95 cases in 2032, reflecting both population growth and projected improvements in maternal health outcomes over time (see Table 6).

**Table 6:** Estimate of post-abortion complications

Year	2023	2024	2032
Total reproductive age female population	14,907,057	15,057,719	16,319,144
Pregnancy rate at 17.5 births per 1000 people	260,874	263,510	285,585
Unintended pregnancies (28.40%)	74,088	74,837	81,106
Abortion count of all pregnancies (29%)	75,653	76,418	82,820
Abortion count of unintended pregnancies	45,194	45,650	49,475
Risk of post-abortion complications	0.16%	0.10%	0.07%
Estimated number of post-abortion complications	193	195	95

These estimates suggested that the Facility Kit could serve a critical role in meeting post-abortion care needs among women in disaster-prone districts.

#### Facility Kit: number of persons served per kit

Most health facilities in Cox’s Bazar District were reported to be adequately equipped to provide post-abortion care services under normal conditions, as per the FGD and the key informant consultant with health managers; however, service delivery gaps persist in hard-to-reach areas, particularly during disasters. Findings from the survey and key informant interviews indicated that the anticipated need for a Facility Kit in many district health facilities was limited to a maximum of 5 affected individuals (see the section on survey findings below). In response to these findings, and following consultation with the TAG, the concept of a small, modular Facility Kit was developed, designed to be procured and pre-positioned at the Union level to meet these limited yet critical needs.

These design considerations were informed by estimates of the PAC-affected population in selected Districts, complemented by survey findings, FGDs, key informant interviews, and ongoing technical consultations. Based on this evidence, the required capacity of the Facility Kit was determined to range from 5 to 30 affected persons, to accommodate variation in facility-level capacity; accordingly, three different kit sizes were developed.

- Small Kit for 5 affected persons—suitable for Union Health and Family Welfare Centres
- Medium Kit for 15 affected persons – suitable for Upazila Health Complex
- Large Kit for 30 affected persons – suitable for Upazila Health Complex and Mother and Health Welfare Centres.

The option to procure individual components/packages (medicines, equipment, consumables) or combinations was also conceived to offer flexibility to health facilities. Additionally, two types of Facility Kits were introduced, in line with national guidelines: (i) PAC only, and (ii) PAC with MR.

#### Mobile Kit: number of persons served per kit

Surveys of 30 emergency shelters found minimum capacities of 50–80 persons and maximum capacities ranging from 1,300 (plain) to 2,500 (coastal). Average shelter stays during recent disasters, including Cyclone Mocha (2023), Cyclone Sitrang (2022), Cyclone Hamoon (2023), and the floods (2023), were 1.47 days, varying by terrain: plain (1.75), hilly (1.0), and coastal (1.17). The key informant consultants also emphasised that evacuees stay between one and five days. This opinion is consistent with the survey findings (see Table 7).

**Table 7:** Duration of the evacuees' stay at the shelter during the most recent disaster

Survey data					Key informant consultation
Status	Total	Plain	Hilly	Coastal	The evacuees stayed between one and five days. As soon as the water recedes, evacuees return to their homes
Number of shelters	30	16	2	12	
Mean	1.47	1.75	1.00	1.17	
Maximum	10	10	1	2	
Minimum	1	1	1	1	

Women’s shelter use also varied, with coastal shelters accommodating higher numbers (mean 185). Approximately 33% of shelters provided primary treatment for pregnant women, with all referring patients to nearby health facilities (Union Health and Family Welfare Centres). The average distance to the nearest facility was 2.24 km. Variations exist across different terrains, with coastal areas generally exhibiting a higher average distance (3.29 km) compared to plain (1.53 km) and hilly areas (1.55 km). Only one coastal emergency shelter reported having Family Welfare Visitors, Family Welfare Assistants, and midwives deployed during disasters.

During the FGD, participants stated that they would immediately go to their station to provide emergency health services whenever they were notified of an upcoming disaster. They also travel between different shelter centres to meet the healthcare needs of evacuees and remain active until the end of the disaster. Although this finding is inconsistent with the survey data (only one shelter in the coastal area had a health worker deployed during the most recent disaster), it is likely that emergency mobile service practices exist, thus increasing the feasibility of the Mobile Kit.

Based on these findings and in consultation with the TAG, the Mobile Kit was designed to serve up to five affected individuals. Participants in the FGDs and key informant consultations recommended that the Kit be positioned at emergency shelters and Union Health and Family Welfare Centres or carried in backpacks by service providers. As such, an accessory – a waterproof, portable backpack for service providers – was introduced for the Mobile Kit box.

### Estimated total and itemised unit cost required to produce one complete Facility and Mobile Kit prototype (RQ2)

The estimated price for the Kits was determined from two sources: the open market price and RHSTEP.

#### Open market price

The content price for the Facility and Mobile Kits was determined based on the current market prices, including any value-added taxes required for procurement under the government or institutional framework. See Tables 8 and 9**,** which show the costs to produce the Facility Kit for PAC only and for PAC and MR. The production cost for the Mobile Kit is BDT 24,070 (approx. USD 206).

**Table 8:** Cost of Facility Kit based on open market (retail) prices – PAC only

Size/Component price	Medicines		Equipment		Consumables		Total Price^a^	
	BDT	USD	BDT	USD	BDT	USD	BDT	USD
Small	17,447	149	113,250	968	11,590	70	142,287	1,216
Medium	51,218	438	113,250	968	13,090	79	177,558	1,518
Large	107,941	923	133,950	1,145	16,260	98	258,151	2,206

^a^Exchange rate is subject to change. This price was estimated at 1 USD = 117 BDT

**Table 9:** Cost of Facility Kit based on open market (retail) prices – PAC & MR

Size/Component price	Medicines		Equipment		Consumables		Total Price^a^	
	BDT	USD	BDT	USD	BDT	USD	BDT	USD
Small	21,347	182	113,250	968	11,590	99	146,187	1,249
Medium	62,518	534	113,250	968	13,090	112	188,858	1,614
Large	130,141	1,112	133,950	1,145	16,260	139	280,351	2,396

^a^Exchange rate is subject to change. This price was estimated at 1 USD = 117 BDT

#### RHSTEP price

As mandated by the DGFP’s Comprehensive MR and PAC Services Guidelines, RHSTEP complies with all national regulatory requirements, including those applicable to procurement activities related to the Kit. RHSTEP packaged one of each size for PAC and MR. The consolidated weight of the Facility Kit-PAC and MR is 50 kg for the small size, 55 kg for the medium size, and 73 kg for the large size. Kits are packaged in waterproof cardboard boxes and comply with national procurement regulations. Production costs, based on RHSTEP supplier estimates, are presented in Tables 10 and 11 for PAC-only and PAC & MR versions.

**Table 10:** Cost of Facility Kit – PAC only

Size/Component price	Medicines		Equipment		Consumables		Total Price^a^	
	BDT	USD	BDT	USD	BDT	USD	BDT	USD
Small	11,000	95	39,112	334	12,336	105	62,547	535
Medium	31,980	273	39,437	337	12,957	111	84,374	721
Large	69,745	596	53,948	461	14,131	121	137,823	1,178

^a^Exchange rate is subject to change. This price was estimated at 1 USD = 117 BDT

**Table 11:** Cost of Facility Kit – PAC & MR

Size/Component price	Medicines		Equipment		Consumables		Total Price^a^	
	BDT	USD	BDT	USD	BDT	USD	BDT	USD
Small	12,902	103	39,112	334	12,336	105	63,540	543
Medium	34,815	298	39,437	337	12,957	111	87,209	745
Large	75,273	643	53,948	461	14,131	121	143,351	1,225

^a^Exchange rate is subject to change. This price was estimated at 1 USD = 117 BDT

The Mobile Kit weighs 5.4 kg and costs BDT 9,612 (USD 82). It is packaged in a waterproof cardboard box and includes an additional backpack for easy portability.

#### Additional costs

Transport costs are estimated at 8–12% of the Kit price, with an additional 8% procurement and packaging fee. Final costs may vary depending on market fluctuations and applicable value-added taxes.

#### Cost variations

Comparative analysis showed that retail procurement of individual items would increase costs by 37–95% compared to supplier-based procurement. On the other hand, bulk purchasing offers potential for further cost reduction.

### Effectiveness of the kit localisation compared to the standard IARH Kit 8 (RQ3)

Currently, the IARH Kit 8 is the only other Kit available globally for PAC during disasters and crises and therefore serves as a benchmark. It should be noted, however, that the comparison is not a straightforward one due to the differences in the use case for the Facility and Mobile RH Kits, which were developed in response to climate-related disasters, compared to the IARH Kit 8, which is meant for an acute phase of an emergency situation in general. The findings for the six dimensions of comparison are below.

#### Use cases

Climate-related disasters tend to follow a pattern each year, which makes pre-positioning of Facility and Mobile RH Kits more feasible alongside other preparedness measures, such as disaster warning systems. IARH Kits, on the other hand, prioritise responsiveness and rapid deployment at the onset of an emergency, including war and conflicts, which could be more difficult to anticipate. As such, pre-positioning of IARH Kits is advised only as a last resort due to the risk of resource waste.

#### Procurement and logistics

As pre-positioning of the Facility and Mobile RH Kits is feasible and encouraged, the production, procurement, transportation, and warehousing could be planned in advance of the disaster season. Kit components can be sourced domestically or near the disaster sites, rather than a centralised global system used for IARH Kits. In addition, planning and pre-positioning would help minimise the need for airfreight (e.g., IARH Kits being flown from Copenhagen), which is often required for unforeseen emergency situations.

#### Flexibility

IARH Kit 8 comes in one size and is intended for 60 affected persons. In a smaller country with pockets of hard-to-reach areas and a scattered network of health facilities, the need for each facility may vary, making it not always cost-effective to purchase a single standard-sized kit for each facility. Facility Kits are available in three sizes – small, medium, and large—for 5, 15, and 30 affected persons, respectively, providing greater flexibility for the main use case. The flexibility is also enhanced with the modular design, as medicine, equipment, and consumable supplies can be procured independently, subject to needs assessment. In addition, the Mobile RH Kit is portable and sufficient for the first-line care of 5 affected persons in hard-to-reach areas.

#### Regulatory compliance and adaptability

Facility and Mobile RH Kits have been developed to comply with Bangladesh’s Comprehensive MR and PAC Service Guidelines, whereas the IARH Kit 8 cannot do so due to its design and purpose. Adaptation to other contexts, such as other LMICs with significant risk of climate-related disasters, could be possible based on the localisation process carried out in Bangladesh.

#### Cost and value for money

Cost comparison is arguably the least straightforward element due to the differences in intended use cases. IARH Kits are suitable for larger facilities and share some common components. For example, a steriliser is often required for PAC but not included in IARH Kit 8, as it is in Kit 6 A (Clinical Delivery Assistance). A similar situation applies to other medicines and supplies that are important for patient care but not strictly or exclusively related to PAC. As a result, the Facility RH Kit contains nearly twice as many items as the IARH Kit 8 (67–69 vs 38), including contraceptives, pain management, hydration, vitamins, PPE, hygiene supplies, and sterilisation equipment. Instruction guidelines in English and the local language are also included. A more comprehensive cover for the use case is reflected in the prototype costs (USD 745 for medium and USD 1,225 for large), which are not necessarily lower than those of the IARH Kit 8 in absolute terms (USD 1,139–1,344).

Therefore, other factors contributing to value for money should also be considered, such as the type of emergency, the pattern of displacement, the general setup and locations of health facilities, transportation costs, and the specifics of local regulations, among others.

The key differences are summarised in Table 12.

**Table 12:** Comparative overview between IARH Kit 8 and Facility Kit

Dimensions	IARH Kit 8	Facility RH Kit – PAC & MR, PAC
Use cases	• Designed for generic crisis situations	• Designed for climate-related disasters, but may also be used in humanitarian crises and health emergencies • Specific design considerations for hard-to-reach areas
Procurement and logistics	• Purchase from Copenhagen • Pre-positioning is only advised as a last resort due to the risk of significant waste	• Components sourced, assembled, and posted domestically or regionally to speed up response time and lower cost • Planning and pre-positioning are recommended
Flexibility	• One single standard size	• Available in multiple sizes • Modular components (medicine, equipment, consumables) are available separately
Regulatory compliance	• Not specific to local regulations	• Compliant with the Comprehensive MR and PAC Service Guidelines of Bangladesh
Cost	Price (excluding transportation and overhead costs): • USD 1,139	RHSTEP Price (excluding transportation and overhead costs): PAC & MR • Small – USD 543 • Medium – USD 745 • Large – USD 1,225 PAC only • Small – USD 535 • Medium – USD 721 • Large – USD 1,178
Value for money based on content comparison	• Number of items (excluding documents): 38 • Core items (medicines & equipment) for the care of miscarriages and PAC • IEC materials in English and French • Length of intervention period: 3 months	• Number of items (excluding documents)^a^: 67 (PAC) or 69 (PAC & MR) • Core items for IARH Kit 8 purpose, plus: • Medicines – Contraceptives, pain management, hydration, supplements, topical medicine^b^ • Equipment – Sterilization^c^, PPE & hygiene^d^ • IEC materials in English and Bangla (or local language) • Instruction guidelines in English and Bangla • Two-Step Checklist Tool to help assess the appropriate size and contents of the Kit required by the user and customer • Length of intervention period: 3 months

^a^Total number comprises: Medicine (23 PAC/25 PAC & MR); Equipment (32); Consumables (12)

^b^These items were included through intensive consultation during the protocol phase of this study. They are available in the IARH Kits 1,4,5,6B,9

^c^This item is available in the IARH Kit 6A

^d^These items are available in the IARH Kit 6B

## Discussion

### Estimated number of affected persons for each kit

The estimates presented highlight both the scale of reproductive health needs in selected disaster-prone districts and the importance of tailoring kit design to local contexts. Although the absolute number of complications appears modest at the aggregate level (195 in 2024 and 95 in 2032), these figures reflect critical, time-sensitive cases concentrated in disaster-prone, hard-to-reach settings where access to comprehensive PAC services is frequently disrupted. In such contexts, even a small number of unmanaged post-abortion complications can result in disproportionate levels of maternal morbidity and mortality. As such, the estimates underscore the need for prepositioned, facility-level preparedness solutions, particularly ahead of the monsoon season, to ensure timely and adequate PAC during disasters in Bangladesh. The Facility Kit is therefore conceptualised as a targeted response to the estimated burden of post-abortion complications in vulnerable districts, rather than as a high-volume service delivery model.

However, this assumption rests on the premise that governments in Bangladesh and other LMICs prioritise reductions in maternal mortality and PAC-related morbidity, and that sufficiently trained service providers are available to deploy and manage the kits. Where such prioritisation, workforce capacity, or institutional support is lacking, the potential of the Facility Kit to strengthen primary health care preparedness and disaster resilience may not be fully realised. In these settings, opportunities to embed PAC-focused disaster readiness within routine health system functioning risk being missed.

These concerns are particularly salient in Bangladesh, where the health system continues to face governance challenges and chronic underinvestment [62]. Health infrastructure and workforce density remain low, with one hospital bed per 1,196 people in 2017 and one registered physician per 1,581 people in 2018. Basic service readiness indicators remain weak, while public health expenditure has remained below 1% of GDP for more than two decades [62]. At the same time, key reproductive health indicators—including total fertility rate (TFR), contraceptive prevalence rate (CPR), and unmet need for family planning—have shown limited improvement over the past decade [38, 63]. The rising rate of contraceptive discontinuation within 12 months—from 30% in 2014 to 37% in 2017–18 [64], further suggests sustained vulnerabilities across the reproductive health continuum of care, which may intensify demand for PAC during disasters.

Taken together, these findings suggest that estimates of the number of affected persons served by the Facility Kit should not be interpreted as fixed-capacity metrics. Rather, they reflect the interaction between minimum service quality thresholds required to ensure safe and timely post-abortion care and underlying health system readiness, both of which are central to building resilient reproductive health services in disaster-prone settings such as Bangladesh.

### Estimated cost

The cost of one Facility Kit and one Mobile Kit supplied by RHSTEP, despite sourcing the contents locally, is not inexpensive. The cost of the Facility Kit (excluding additional costs and price variations) is nearly on par with that of the IARH Kit 8. As only a single Facility and Mobile Kit were prototyped, these cost estimates are conservative. In comparison, costs calculated using open-market retail prices were substantially higher, ranging from 37 to 95%. Procurement through an established supplier such as RHSTEP, therefore, resulted in significant cost savings, with further reductions anticipated through bulk purchasing, reflecting a more realistic procurement scenario for disaster preparedness operations. The lead author is currently working with a pharmaceutical company to obtain more accurate pricing.

Beyond nominal unit cost comparisons, the estimated cost of the Facility and Mobile Kit should be interpreted in relation to its functional scope and contextual suitability. While the Facility Kit's cost is comparable to that of IARH Kit 8, its novelty lies in its localisation to align with Bangladesh’s MR and PAC Service Guidelines, as well as the specific needs of disaster-prone, hard-to-reach areas. Designed for integration into the PHC system, the Kit prioritises safe and timely PAC delivery during emergencies, particularly through prepositioning at primary and secondary health facilities ahead of the monsoon season. This is especially relevant given that Bangladesh experiences annual rainfall-induced flooding, with approximately 80% of total rainfall occurring during the monsoon period [65].

Local sourcing and context-specific configuration may therefore offer enhanced operational value, including improved usability, faster deployment, and reduced reliance on international supply chains. From this perspective, cost parity with IARH Kit 8 does not necessarily indicate reduced affordability, but rather reflects the resources required to ensure guideline-compliant PAC provision in fragile and high-risk settings.

Moreover, although the upfront unit cost may appear relatively high, the strategic pre-positioning of the Kits in high-risk districts has the potential to generate downstream savings by preventing severe PAC that would otherwise require referral, hospitalisation, or emergency surgical intervention. As such, the cost of the Facility and Mobile Kit is more appropriately assessed in terms of affordability, budget impact, and health system efficiency rather than unit price alone, particularly in settings where delayed or disrupted PAC services contribute to preventable maternal morbidity and mortality.

### Localised item composition of the Facility Kit

Although a comparative analysis was undertaken between the IARH Kit 8 and the Facility Kit, these kits are best understood as complementary rather than substitutive. The IARH Kits, including Kit 8, form a core component of the MISP for sexual and reproductive health in humanitarian settings globally. By contrast, the Facility and Mobile Kits developed and prototyped in this study are locally tailored preparedness and response tools that align with Bangladesh’s Comprehensive MR and PAC Service Guidelines. Their broader purpose is to address context-specific primary healthcare needs of women and adolescent girls in disaster-prone, rural, and hard-to-reach facilities, while strengthening the health system's disaster resilience at the primary care level.

MR or safe abortion remains a legally and socially sensitive issue in Bangladesh. While abortion is illegal, MR is permitted within set time limits of up to 12 weeks. Globally, legal frameworks for abortion and PAC vary considerably, with approximately 60% of women (1.2 billion) of reproductive age living in contexts where abortion is broadly legal and 40% (801 million) living under restrictive laws [66]. The Facility Kit reflects this diversity by offering both a combined MR & PAC version and a PAC-only version, allowing flexibility for adoption across different regulatory and programmatic contexts. Moreover, national restrictions on the availability of antibiotics, pain management, contraceptives, and emergency medicines require careful alignment with country-specific regulations. The modular design of the Facility Kit allows its contents to be updated in line with evolving national MR and PAC guidelines, reinforcing its adaptability within regulated health systems.

The Facility and Mobile Kits are designed to respond to the resource-constrained realities in which primary healthcare facilities operate in disaster-prone, hard-to-reach areas of Bangladesh. Their development was informed by service delivery challenges observed during the COVID-19 pandemic, which highlighted the importance of infection prevention, occupational safety, and service continuity. Accordingly, the Kits include personal protective equipment such as aprons, gloves, goggles, masks, plastic gum boots, and waterproof coats, alongside disinfectants (e.g. povidone-iodine) and essential consumables including cotton wool, sanitary napkins, biohazard bags, antiseptic solutions, and sterile surgical gloves [15]. Additional items, such as draw sheets, plastic bed covers, and portable lighting, support women’s privacy and dignity and ensure safe clinical practice during procedures. While primarily conceived for natural hazard-related emergencies, the Kits are also applicable to other public health emergencies in which routine service delivery is disrupted.

Furthermore, the Kits include options for capacity-building training in MR and PAC for clinical health workers, as well as community awareness-raising workshops for community-level SRH service providers. Administration of the Kits is intended to be undertaken by government-mandated service providers, ensuring alignment with national service delivery protocols and regulatory requirements. However, PHCs in hard-to-reach locations frequently experience acute shortages of trained health personnel and adequate human resources [13, 22, 67]. In the absence of a skilled workforce, the Facility and Mobile Kits cannot be effectively pre-positioned, deployed, or administered. Bangladesh’s pluralistic health system, which includes MR and PAC services, presents opportunities to address these gaps. Several non-governmental organisations, such as the Family Planning Association of Bangladesh, RHSTEP, Marie Stopes, and Pathfinder, already deliver MR and PAC services and could potentially host or support the deployment of the Kits in areas where government-run PHC facilities face capacity constraints.

Last but not least, the Kits design centres on the needs of vulnerable women and girls of reproductive age. Severe anaemia, micronutrient deficiencies, and malnutrition among mothers and adolescent girls remain significant public health concerns in Bangladesh [68]. Accordingly, the Kits include supplements such as calcium tablets, iron–folic acid, and zinc tablets. They also contain topical medicines—benzyl benzoate lotion and miconazole cream—to mitigate fungal infections following floods. Disaster-associated fungal infections are increasingly recognised and typically occur during the impact phase, due to inhalation or cutaneous inoculation with environmental spores [69]. These items are not available in the IARH Kit 8. The Facility Kit additionally includes emergency medicines such as tranexamic acid (IV injectable) and an antiemetic (injection metoclopramide) to manage complications and provide immediate care, including for survivors of gender-based violence such as rape.

Taken together, the localised item composition of the Facility Kit demonstrates closer alignment with Bangladesh’s MR and PAC guidelines and the practical realities of service delivery in disaster-affected primary healthcare settings. Rather than duplicating the role of IARH Kit 8, the Facility and Mobile Kits aim to extend preparedness and response capacity by addressing broader reproductive health needs before, during, and after disasters, thereby supporting continuity of care and strengthening health system-level resilience in hard-to-reach areas.

## Conclusion

This paper presents findings from the prototype development of the Facility and Mobile RH Kits, two localised novel solutions designed to provide PAC and safe abortion or MR during disasters and crises.

The prototypes represent a significant innovation in disaster preparedness for SRH issues related to PAC and safe abortion or MR. Unlike existing kits (IARH Kit 8 and Sterimed Group’s Manual Vacuum Aspiration set), which are often generic and not tailored to crisis contexts, the Facility and Mobile RH Kits were designed through local stakeholder engagement and evidence-based research on crises and needs in Bangladesh. They integrate locally approved medicines and equipment, ensuring compliance with national guidelines while maintaining portability and rapid deployment features. The Mobile Kit addresses last-mile delivery challenges by enabling trained providers to reach women in remote and hard-to-reach areas where health facilities are inaccessible. We posit that this localised innovation has the potential not only to improve access to safe abortion or MR and PAC care during disasters and crises but also to strengthen health system resilience by increasing the facility capacity and embedding disaster risk reduction principles into reproductive health service delivery. By bridging gaps in availability, affordability, and acceptability, these Kits offer a scalable model for LMICs facing recurrent disasters.

It is therefore recommended that these Kits receive support from the Government of Bangladesh and international donors (e.g., UNFPA, IPPF) to evaluate their effectiveness in improving PAC and safe abortion or MR management in hard-to-reach and humanitarian settings across Bangladesh. Additionally, it would be useful to replicate the process of localising the Kits in other countries and regions, as well as to gather evidence from piloting their use.

## Data Availability

The raw survey data supporting the findings of this study are available upon request from the authors. The itemised lists for both Facility and Mobile RH Kits are currently confidential and therefore subject to embargo. However, detailed descriptions of the Facility and Mobile Kit contents are available only upon request. Ethical approval for this study was obtained in 2024 from the University of Leicester’s Research Ethics Committee (reference: 43,496-nsrb1-ss/bu:m'ing,innov',str'gy&ops,dept) and the University of Dhaka’s Institute for Health Economics Institutional Review Board (reference: HE/IRB/DU/70/2023/Final).

## Footnotes

1 In addition, the lead author has prior experience working in this district and has established professional networks in Cox’s Bazar. Consequently, it was considered a suitable district for gathering the necessary evidence to inform the prototype of the Kits.

2 https://www.shwapno.com/

3 https://chaldal.com/
